# A high-performance complementary inverter based on transition metal dichalcogenide field-effect transistors

**DOI:** 10.1186/s11671-015-0827-1

**Published:** 2015-03-10

**Authors:** Ah-Jin Cho, Kee Chan Park, Jang-Yeon Kwon

**Affiliations:** School of Integrated Technology, Yonsei University, 85 Songdoguahak-ro, Incheon, 406-840 Korea; Yonsei Institute of Convergence Technology, 85 Songdoguahak-ro, Incheon, 406-840 Korea; Department of Electronic Engineering, Konkuk University, 120 Neungdong-ro, Seoul, 143-701 Korea

**Keywords:** Transition metal dichalcogenide (TMD), CMOS inverter, 2-Dimensional material

## Abstract

For several years, graphene has been the focus of much attention due to its peculiar characteristics, and it is now considered to be a representative 2-dimensional (2D) material. Even though many research groups have studied on the graphene, its intrinsic nature of a zero band-gap, limits its use in practical applications, particularly in logic circuits. Recently, transition metal dichalcogenides (TMDs), which are another type of 2D material, have drawn attention due to the advantage of having a sizable band-gap and a high mobility. Here, we report on the design of a complementary inverter, one of the most basic logic elements, which is based on a MoS_2_ n-type transistor and a WSe_2_ p-type transistor. The advantages provided by the complementary metal-oxide-semiconductor (CMOS) configuration and the high-performance TMD channels allow us to fabricate a TMD complementary inverter that has a high-gain of 13.7. This work demonstrates the operation of the MoS_2_ n-FET and WSe_2_ p-FET on the same substrate, and the electrical performance of the CMOS inverter, which is based on a different driving current, is also measured.

## Background

With the advent of graphene, one of the most studied 2-dimensional (2D) materials, layered materials attracted much attention due to their unique and outstanding electrical, mechanical, and optical characteristics [1,2]. In spite of having an extremely high mobility of 15,000 cm^2^/Vs and of being easy to scale down, the absence of an inherent band-gap limits the potential for graphene to be implemented in logic circuits [3].

Transition metal dichalcogenides (TMDs) are a family of 2D materials and are considered to be good candidate materials that can overcome the limitations of graphene. TMDs follow the formula *MX*_2_, where *M* is a transition metal and *X* stands for a chalcogen (S, Se, Te, etc.). TMD consists of atomic layers that have very weak van der Waals forces binding them, and they usually have a band-gap of 1.1 to 1.9 eV [4]. Such properties enable TMDs to be used as channel materials in high-performance field-effect transistors (FET) [5]. Of the various TMD materials available, MoS_2_ and WSe_2_ have been individually reported to be suitable channel materials for n-type and p-type transistors. The MoS_2_ n-FET demonstrated a mobility of approximately 200 cm^2^/Vs, and an effective hole mobility for WSe_2_ p-FET of up to approximately 250 cm^2^/Vs was also reported [6,7].

A complementary metal-oxide-semiconductor (CMOS) inverter is a fundamental unit for the logic elements of a circuit. In order to fabricate a CMOS inverter, both p-type and n-type transistors are necessary [8]. In comparison to a resistive-load inverter, a complementary inverter presents outstanding properties in terms of having low power consumption and a large noise margin [9].

There have been several studies trying to fabricate complementary inverter using TMDs. Huang J *et al*. [9] have reported the first CMOS inverter using TMD, which was fabricated with MoS_2_ and carbon nanotube as channel materials. After this report, researchers tried several other p-channel materials like Bi_2_Sr_2_Co_2_O_8_ [10] or phosphorene [11], and fabricated complementary inverter with a combination of MoS_2_. Such reports possess significance in that both n-channel and p-channel are layered materials, but still, the electrical performances of the devices were insufficient. The first fully TMD-based CMOS inverter was achieved by Tosun M *et al*. [12] by utilizing one material of WSe_2_. For p-channel, pure WSe_2_ was used and for n-channel, K-doped WSe_2_ was used. The resulting device exhibited high peak gain of approximately 12, but due to the instability of K doping in air, its performance tends to degrade as operation time goes on. Recently, Das S *et al*. [13] have reported WSe_2_-based complementary inverter showing a gain of over 25. As they carefully engineered the threshold voltage of both n-FET and p-FET which constitutes the CMOS inverter, the resulting device property was remarkable.

Here, we report on a fully TMD-based CMOS inverter which is comprised of a MoS_2_ n-FET and a WSe_2_ p-FET. The peak gain for our complementary TMD inverter reaches a value of over 13 at a driving voltage of 2 V. We used two different intrinsic n-type and p-type TMDs individually, and achieved a reasonable performance of MoS_2_-/WSe_2_-based CMOS inverter which could be a great potential of TMDs in logic applications.

## Methods

Several layers of each TMD were obtained from commercially available bulk MoS_2_ (429ML-AB, SPI Supplies Inc., West Chester, PA, USA) and WSe_2_ (NS00182, Nanoscience Instruments Inc., Phoenix, AZ, USA) crystals via mechanical exfoliation using an adhesive tape. A highly-doped silicon substrate with a 30-nm-thick atomic layer deposition (ALD) Al_2_O_3_ was used to fabricate the complementary TMD inverter. Multi-layer MoS_2_ was transferred onto half the area of one substrate, and WSe_2_ was transferred onto the other half. Then, 200 μm × 200 μm square electrode arrays were fabricated for each channel material through a conventional lift-off process. In order to form ohmic contacts, the 30 nm Ti and 30 nm Pt were individually used as the metals for the electrical contacts of the n-FET and p-FET by considering the work functions of MoS_2_ and WSe_2_. After fabricating the n-FETs and p-FETs on one substrate, a Keithley 4200 parameter analyzer was used to measure the electrical performance of the MoS_2_ and WSe_2_ FETs.

Based on the results of the measurements, we chose several sets of n-FETs and p-FETs that showed a reasonable performance. Finally, a 50-nm-thick Ti connecting line was fabricated between the selected MoS_2_ and WSe_2_ FETs through a lift-off process, and it was followed by electron-beam deposition. After the CMOS inverter structure was fabricated, the devices underwent annealing for 2 h in a tube furnace at 200°C with an N_2_ atmosphere in order to decrease the contact resistance between the channel and the metal electrode. A Keithley 4200 parameter analyzer was used to measure the electrical performance of the MoS_2_/WSe_2_ CMOS inverter. The measurements were carried out using four probes to plot the graph for the input and output voltage of the inverter. The driving voltage (*V*_DD_) was applied to the source of the p-FET, and ground was connected to the source of the n-FET. The input voltage was applied to the bottom gate of MoS_2_ FET and WSe_2_ FET at the same time by sweeping from −5 to 0 V, and the output voltage was recorded by measuring at the connecting line for both drains.

## Results and discussion

**Figure 1:**
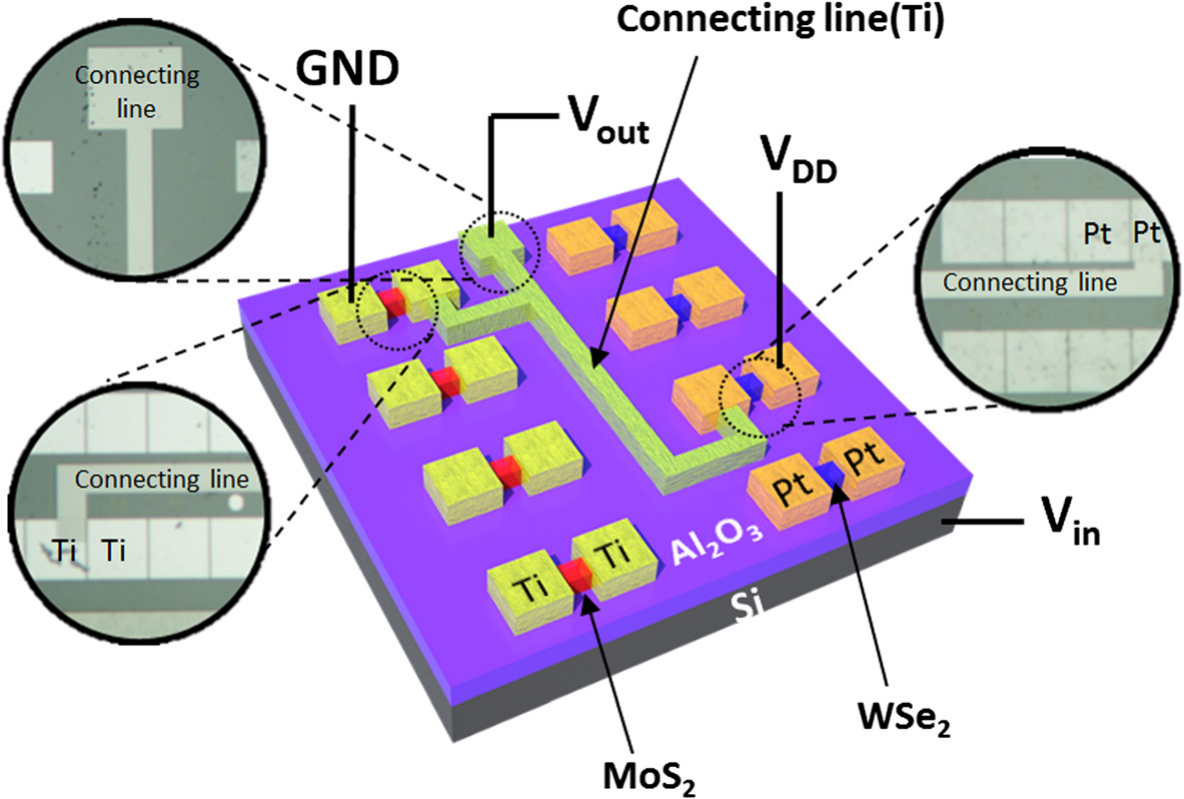
**Device structure with electrical connections.** Schematic illustration and optical microscopy image of a MoS_2_/WSe_2_ complementary inverter with electrical connections that were used to measure the electrical characteristics.

**Figure 2:**
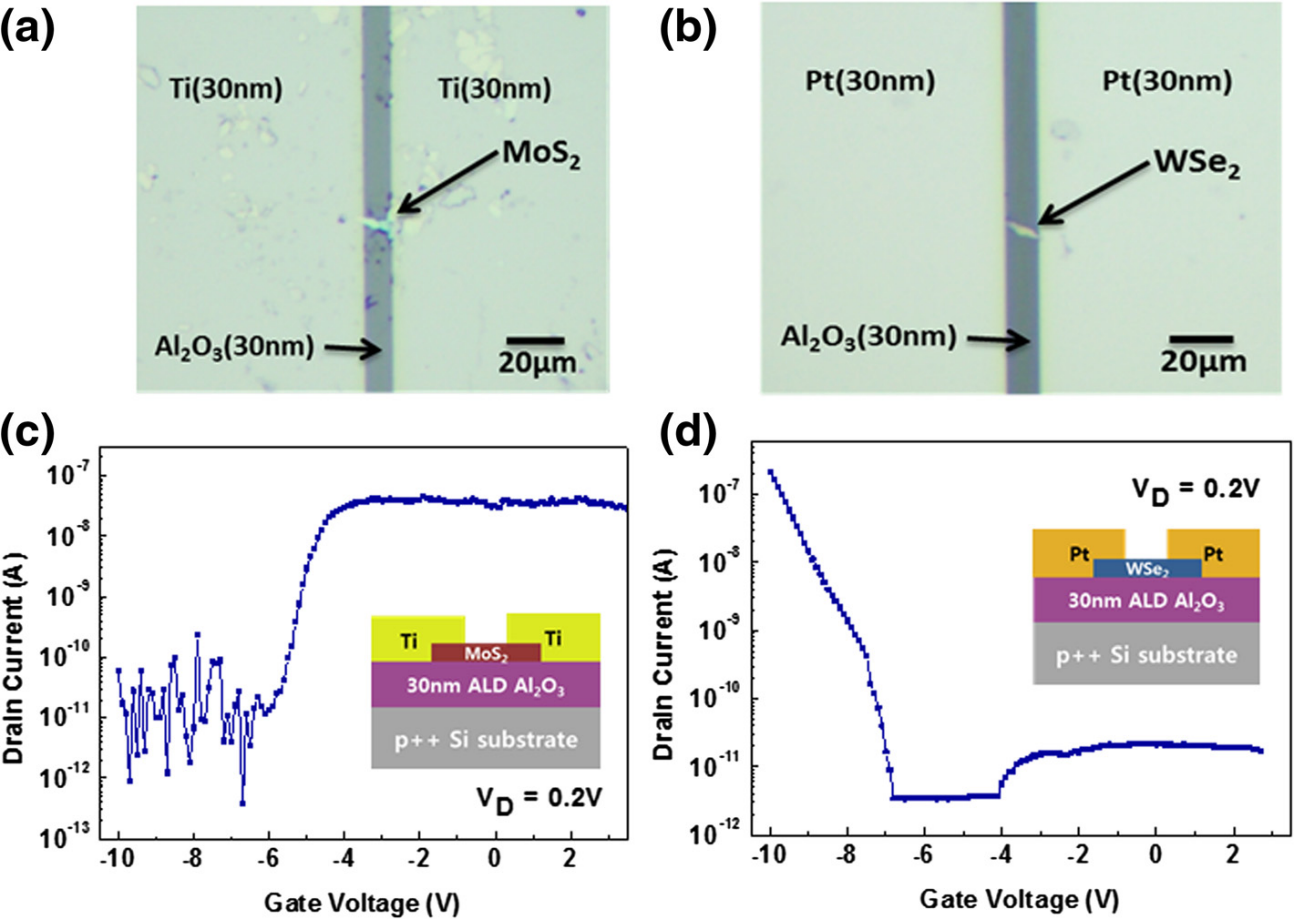
**Optic images and transfer characteristics of n-** **FET and p-** **FET.** Optical microscopic image of **(a)** the MoS_2_ n-FET and **(b)** the WSe_2_ p-FET that was employed to fabricate the TMD CMOS inverter. The transfer characteristics of **(c)** the MoS_2_ n-FET and **(d)** the WSe_2_ p-FET at a drain voltage of 0.2 V.

Figure 1 depicts the overall structure of the TMD CMOS inverter that contains both an n-FET and a p-FET with TMDs as channel materials. We used multi-layer MoS_2_ for the n-type channel and multi-layer WSe_2_ for the p-type semiconducting material (Figure 2a,b). Ti was chosen as the metal electrode for the MoS_2_ FET since that metal is known to have a small work function, and it is therefore easier to form an ohmic contact with MoS_2_ [4]. On the other hand, WSe_2_ has unique characteristic in that a simultaneous injection of both holes and electrons can easily occur, resulting in ambipolar transport [5,7]. In order to meet our goals for fabricating the p-type FET, a metal with a large work function had better be used for the electrode. Therefore, we chose Pt for the WSe_2_ FET since Pt has a work function of 5.12 to 5.93 eV.

The transfer characteristics were measured in order to verify the electrical performance of the FETs. Figure 2c,d presents the resulting transfer curves for the n-FET and p-FET. The MoS_2_ FET shown in Figure 2c has 6-μm channel width and 10-μm channel length. It shows clear n-type characteristics with an *I*_on_/*I*_off_ ratio of approximately 10^5^. As the gate voltage increases to be higher than the threshold voltage (−5.23 V), the drain current drastically increases up to approximately 10^−7^ A. The field-effect mobility of the MoS_2_ FET derived from the transfer characteristic is found to be 0.86. The WSe_2_ FET presents p-type characteristics caused by the hole injection. The drain current tends to increase sharply, up to approximately 10^−7^ A, under a gate bias that is less than its threshold voltage, −8.79 V. This device with 2-μm channel width and 10-μm channel length exhibits *I*_on_/*I*_off_ ratio of approximately 10^4^ and mobility of 8.87. Both the n-FET and p-FET show a moderate current on-off ratio with a similar on-current level, which is one of the key requirements to work as a complementary inverter.

**Figure 3:**
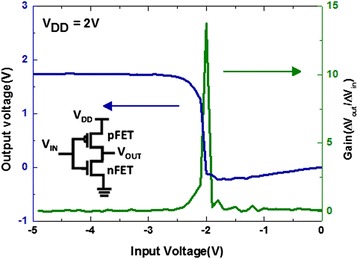
**Voltage transfer characteristics of the inverter.** Voltage transfer characteristics and gain curve of a MoS_2_/WSe_2_ complementary inverter at a *V*_DD_ of 2V.

After sorting MoS_2_ n-FET and WSe_2_ p-FET presenting reasonable electrical performances, CMOS inverter was fabricated with them. The two FETs were connected in series, n-FET drain-by-p-FET drain, with a metal line in order to have them work together as a logic inverter. As shown in Figure 1, the electrical connection was configured to measure the performance of the completed complementary TMD inverter. Four probes were used to apply the voltage and to record the output voltage. A constant bias of *V*_DD_ = 2 V was applied, and the relation between the input voltage and the output voltage was measured as shown in Figure 3. The voltage transfer characteristic in Figure 3 was measured from the TMD CMOS inverter consists of n-FET and p-FET indicated at Figure 2c,d, respectively. While the device received a low-input voltage, the p-type WSe_2_ FET was left in the ‘on’ state and the n-type MoS_2_ FET was in the ‘off’ state. Hence, the output voltage remained high, near *V*_DD_. On the other hand, the n-type MoS_2_ FET was in the on state, and the p-type WSe_2_ FET was in the off state when a high input was applied. As a result, an output voltage of 0 V was measured, which indicates that logic operation from 1 to 0 was performed. Such a voltage transfer curve clearly demonstrates the characteristics of an inverter.

From the input–output voltage plot, the gain curve can be calculated as Δ*V*_out_/Δ*V*_in_. As shown in Figure 3, the peak in the gain curve shows the transition point for the inverter. In our work, the peak gain of the MoS_2_/WSe_2_ complementary inverter reaches 13.7, which is a considerably high value when compared to CMOS inverters based on other 2D material FETs. Table 1 presents a summary of the complementary inverters that have been reported along with their peak gain. Several reports have discussed the fabrication of a CMOS inverter and a pseudo-CMOS inverter (an inverter configuration using two ambipolar transistors rather than one n-FET and one p-FET) based on 2D materials. However, a fully TMD-based inverter has only been discussed by Tosun *et al*. [12] and S Das *et al*. [13] who used n-type- and p-type-doped WSe_2_ as the channel materials. It is thought that the characteristics of the inverter proposed in this work are remarkable since it has been fabricated by using TMDs for both the n-channel and the p-channel, with different channel materials. In addition, a peak gain of over 13 is a relatively high value when compared to not only 2D material-based CMOS inverters but also to those based on oxides or organic materials as well (Table 1). It is considered that the fabrication of such a high-performance inverter in our work was accomplished due to the integration of high-mobility TMD materials and a CMOS configuration.

**Table 1:** **Summary of the CMOS inverter peak gain based on various channels**

**Channel material**				
			**Inverter peak gain**	**Ref**.
	**n**-**type**	**p**-**type**		
2D material	MoTe_2_ (ambipolar)		1.4	[14]
	WSe_2_	WSe_2_	12	[12]
	WSe_2_	WSe_2_	25	[13]
	MoS_2_	Carbon nanotube	1.3	[9]
	MoS_2_	Phosphorene	1.4	[11]
	MoS_2_	BSCO	1.7	[10]
	MoS_2_	WSe_2_	13.7	This work
Silcon	Si-nanowire	Si-nanowire	45	[15]
Oxide	GIZO	SnO	1.7	[16]
Organic	OC_1_C_10_-PPV:PCBM blend (ambipolar)		10	[17]

**Figure 4:**
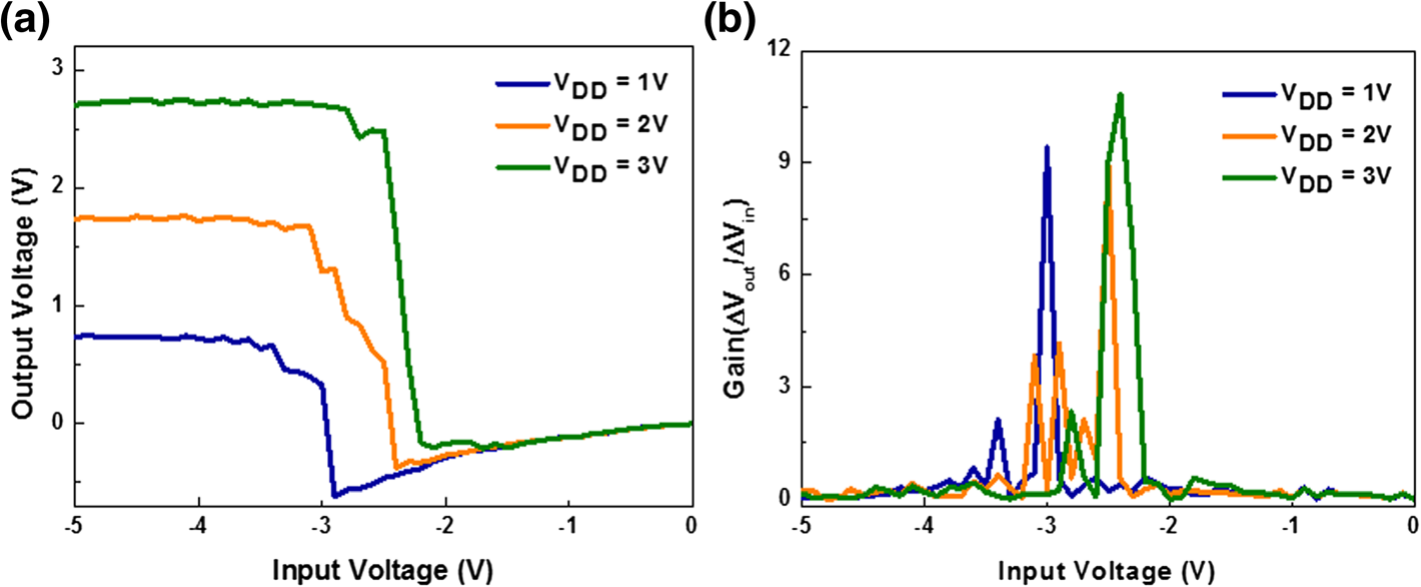
**Electrical properties of the TMD inverter. (a)** Voltage transfer characteristics and **(b)** gain curve for a MoS_2_/WSe_2_ complementary inverter at different driving voltages (*V*_DD_).

In order to further investigate the electrical performance, we changed the driving voltage over three steps, 1, 2, and 3 V, and performed measurements the according voltage transfer characteristics. Figure 4a shows the electrical characteristics of the various voltages used to drive the circuit voltage, and Figure 4b presents the gain plot extracted from the transfer curve. As shown in Figure 4a, our device operates under a negative input voltage. The switching threshold voltage (*V*_M_) of the inverter, which represents the voltage at which the n-FET and p-FET are turned on with the same intensity, is also located in the negative region. These phenomena are all associated with the threshold voltage of the n-FET and the p-FET. Usually the threshold voltage of an n-type transistor has a positive value and that of a p-type transistor has negative value, so the switching threshold voltage is generally located in the range from 0 V to *V*_DD_. However, in our work, both the MoS_2_ FET and the WSe_2_ FET have negative threshold voltages. Accordingly, the equations

1 $$ {V}_{\mathrm{M}}=\frac{V_{\mathrm{Tn}}+\sqrt{\frac{k_p}{k_n}}\;\left({V}_{\mathrm{DD}}+{V}_{\mathrm{Tp}}\right)}{1+\sqrt{\frac{k_p}{k_n}}},\;{k}_{n,\;p}={\left(\frac{W}{L}\right)}_{n,\;p}{\mu}_{n,\;p}{C}_{\mathrm{ox}} $$

can be applied where *V*_Tn_ and *V*_Tp_ are the threshold voltage of the n-FET and p-FET respectively, *W* is width of the channel, *L* is the length of channel, *μ* is the mobility of the FET, and *C*_ox_ is the capacitance of the oxide per unit area. As such, the switching threshold voltage is calculated to be −7.10, −6.37, and −5.64 Vwhen *V*_DD_ = 1, 2, and 3 V, respectively. The positive shift in the voltage transfer curve is due to the shift in the switching threshold voltage, which is also demonstrated in Figure 4a.

As the driving voltage for the inverter changes, the peak gain also changes (Figure 4b). The device tended to show higher gain value as the driving voltage increases. Also, the peak gain appeared at higher input voltage along with the increase of *V*_DD_ from 1 to 3 V. Such phenomena coincide with the well-known operating characteristics of a CMOS inverter.

The electrical property of the TMD CMOS inverter shown in Figure 4 is inferior to the one shown in Figure 3. Such degradation is mainly caused by the environmental effects which are often observed in TMD transistors [18]. As the electrical characteristic under various driving voltage was measured after several days of device fabrication, moisture around the environment might be absorbed to the surface of channel. Uniform encapsulation of the inverter structure will minimize the environmental effects and remain its property under ambient condition.

## Conclusions

In summary, we have fabricated a CMOS inverter by employing an n-type MoS_2_ FET and a p-type WSe_2_ FET to achieve a gain of 13.7. The device clearly operates as a logic inverter by changing the low-input voltage into a high-output voltage and *vice versa*. The working range observed in the negative region is caused by the negative threshold voltage of the n-FET. It is considered that this result is meaningful in that the complementary inverter used two different kinds of TMD materials for the channels, and it exhibits relatively high performance compared to those of devices based on other 2D materials which had been previously reported. This result will provide a step forward towards the fabrication of logic circuits applying TMD materials as a post-Si generation device.
